# Rasch Analysis of the Korean Parenting Stress Index Short Form (K-PSI-SF) in Mothers of Children with Cerebral Palsy

**DOI:** 10.3390/ijerph17197010

**Published:** 2020-09-25

**Authors:** Eun-Young Park, Soojung Chae

**Affiliations:** Department of Secondary Special Education, College of Education, Jeonju University, Jeonju 55069, Korea; eunyoung@jj.ac.kr

**Keywords:** mothers of children with cerebral palsy, Korean Parenting Stress Index Short Form (K-PSI-SF), Rasch analysis

## Abstract

The purpose of this study was to investigate the psychometric characteristics of the Korean Parenting Stress Index Short Form (K-PSI-SF) for mothers of children with cerebral palsy (CP) by using a Rasch analysis. The participants were 114 mothers of children with CP whose ages ranged from 2.79 to 11.90 years. The K-PSI-SF consists of 36 items, with a 5-point Likert scale grading along three subscales (Parent Distress, Parent–Child Dysfunctional Interaction, and Difficult Child). The response data were analyzed, and we determined the item fitness and item difficulty, rating scale fit, and separation index. The results show that two items did not have the required fitness. After these two items were deleted, the means of the 34 items in two of the subscales were statistically different from those of the original 36 items. Our analysis of the item difficulty identified the need to add easier question items. The 5-point Likert scale used in the questionnaire was found to be appropriate. This significance of this study is that it suggested the need to modify item fitness and difficulty level, as it identified the psychometric characteristics of the K-PSI-SF through a Rasch analysis based on the item response theory.

## 1. Introduction

Cerebral palsy (CP), the most common childhood neurological disorder, is defined as an impairment of movement and posture due to immature brain deficit or damage [1]. As a non-progressive lesion, due to abnormal reflex reaction, abnormal muscle tone and lack of proximal stability, normal motor experience is not possible. About 25% to 30% of children with CP become independent adults through rehabilitation treatment [2], but the remaining 70% to 75% require assistance and continuous rehabilitation in adulthood [3].

Consequently, most parents of children with CP must care for their adult children, and their anxiety about their children’s future and consequent parenting stress often lasts relatively longer compared to the parents of ordinary children [4]. Parenting stress is a major factor influencing the development of children in the context of families [5]. Parenting stress was a factor in hyperactivity; aggression due to emotional maladaptive behavior and abuse affected all factors of emotional maladaptive behavior [6]. Parenting stress also affects parenting behavior, which affects emotional and behavioral problems in children; this can create a vicious cycle that increases parental stress [6,7].

In particular, mothers, primary care providers for children with CP, are the most closely related to them, and their role is, therefore, more emphasized. Such mothers often sacrifice their whole lives caring for their children, and they may deprioritize their own happiness as individuals, which may lead to a vicious cycle of psychological distress for children and parents [8].

The parenting stress of parents of children with CP can be higher than that of parents of children with typical development [9]. It is important to reduce the parenting stress of parents of children with behavioral problems [10], children with developmental disabilities and developmentally delayed children [11], and children with chronic illnesses [12]. An intervention program to reduce parenting stress among mothers of children with CP could play a critical role in rehabilitation in this regard; furthermore, studying the intervention effects of such a program after it is developed is essential. A measurement scale with verified validity and reliability is essential for measuring such a program’s effects.

One common tool for measuring parenting stress is the Parenting Stress Index (PSI) [13]. Despite its good psychometric properties, PSI is a long instrument, and the time requirements for evaluation can be enormous. Consequently, a 36-item derivative of the device (short form: PSI-SF) was developed based on a PSI factor analysis representing three-dimensions—Parent Distress (PD), Parent–Child Dysfunctional Interaction (P-CDI), and Difficult Child (DC) [13]. The PSI-SF used data from married mothers of young children (average age 4 years and younger) who are predominantly Caucasian. The correlation between the overall score of the PSI (both full and short version) was very high (0.94) within the group.

The PSI-SF has become one of the most widely used tools for measuring parenting stress across a wide range of families and children [14], including those with disabilities [15], and across 40 different cultures and languages worldwide [16]. In addition to its widespread use in research, PSI-SF is widely used as a clinical tool for identifying parents who need counseling services, thus helping them to conduct family interventions and helping them to evaluate the program. Although the PSI–SF has been applied to a variety of studies [17,18], few investigators have examined its psychometric integrity. Further study across various groups with different characteristics is necessary to confirm its validity. The examination of psychometric properties based on the item characteristics could not be influenced by the characteristics of the study [19].

In Korea, a psychometric validity and reliability study of the Korean Parenting Stress Index (K-PSI) for parents of children with CP indicated that the model’s goodness-of-fit index was similar to that of the standardized K-PSI for normal children, but the reliability of several subscales such as the child subscales, attachment of the parent subscale, competence, and health was not reliable [20]. The item response theory could make it possible to assess the completeness of the test and to remove or modify items within the scale. For that reason, analyses based on item response theory have greater strength than the classical test theories such as exploratory factor analysis (EFA) and confirmatory factor analysis (CFA) [19]. A relatively recent study reported the psychometric properties using item response theory in parents of children with autistic spectrum disorder [21]. The Korean Parenting Stress Index Short Form (K-PSI-SF) is a convenient parenting stress measurement tool because it is useful for objectively and reliably measuring children’s behaviors and their impact on parents’ mental health. However, no studies have examined the psychometric properties of K-PSI-SF in mothers of children with CP based on item response theory. The purpose of this study is to examine the psychometric properties of the K-PSI-SF in mothers of children with CP.

## 2. Materials and Methods

### 2.1. Participants

The participants were 114 mothers of children with CP. Children with CP were receiving rehabilitation treatment at hospitals or community welfare centers or attending schools for children with physical disabilities. The participants in this study were limited to doctor-diagnosed cases of CP. The study was approved by the Research Ethics Board of the Jeonju University on 14 November 2013 (ethical approval code number: Jeonju University IRB-1041042-2013-1).

### 2.2. Measures

This study used the K-PSI-SF [22]. The tool was translated through three stages of work, and a correlation was also calculated among the subscales of K-PSI-SF to confirm convergence validity. Regarding correlations with the Korean-Child Behavior Checklist (K-CBCL) and the Beck Depression Inventory (BDI), the total score was assigned for accredited validity; a different test involving K-PSI-SF scores (between emotional and behavioral disorder groups and normal groups) was conducted to confirm discriminant validity. Table 1 shows the contents of the questionnaire.

K-PSI-SF consists of 36 questions in three subscales: 12 questions from Q1 to Q12 in PD, 12 questions from Q13 to Q24 in P-CDI, and 12 questions from Q25 to 36 in DC. K-PSI-SF was translated into Korean in 2007. Standardized tests were administered to 328 children, and the reliability and validity were conformed [22]. The participants were allowed to select only one answer. The study results showed that the overall reliability of this scale was α = 0.91, the PD reliability was α = 0.90, the P-CDI reliability was α = 0.78, and the DC reliability was α = 0.83. For practice, respondents were asked to read the K-PSI-SF guidelines and write down their personal information. Then, they had to select one answer from the 5-point Likert scale for each of the 36 questions.

### 2.3. Statistical Analysis

We used the rating scale model of the Rasch analysis [23] to evaluate the item fit of the K-PSI-SF. Because the K-PSI-SF does not have different difficult levels for items, we used the rating scale model instead of the partial credit model [24]. We used Winsteps version 3.6 software [25] to examine whether the items of the K-PSI-SF satisfied the basic assumptions of Rasch measurements.

#### 2.3.1. Unidimensionality

To evaluate how well the observed data fit the Rasch unidimensional model and how well each item contributed to defining one common construct, we used the two-item fit mean square (MNSQ) statistics of infit mean squared and outfit mean squared fit index. In the Rasch model, the ideal value of each infit and outfit MNSQ is 1.0, which means that the data fit the Rasch model perfectly. The infit and outfit statistics were presented from Rasch analysis. In this study, if an item fell outside the range of 0.50–1.70 for its infit or outfit MNSQ, it was considered a misfit. People whose response data had an infit value above 2.0 were excluded from the Rasch analysis [26]. There were 12 such people in the study.

#### 2.3.2. Item Difficulty

To examine the item difficulty of the K-PSI-SF, an indication of construct validity, we used a Wright map of the Rasch analysis that allows graphical analysis of persons and items on a map showing the distribution of respondents in the sample. Item difficulty was presented with the item/person map. In the Rasch analysis, items present a hierarchical order of difficulty. Person ability as well as item difficulty along the continuum of logits are expressed as logits. Greater logits imply more difficulty items [26].

#### 2.3.3. Person and Item Separation Index

The person and item separation index is used for describing the reliability of the test in the Rasch analysis. A larger separation index means that a more distinct functioning can be distinguished by the test. A score of above 2.0 on the separation index is a good level of separation. Separation reliability was interpreted in the same way as the Cronbach α [27].

#### 2.3.4. Rating Scale

To examine the suitability of the rating scale (5-point scale) of the K-PSI-SF, we analyzed the category functioning within the Rasch model. The fit values for each rating also provide information about whether each rating works well. Individual fit values for each rating that represents at least 1.5 indicate that the rating scale is not functioning effectively and that the categories need to be integrated later [28]. If the thresholds do not progress linearly, the responses to items are judged to not correspond to the levels of the construct being measured.

#### 2.3.5. Parenting Stress Level

A paired *t*-test was performed to examine the difference in the K-PSI-SF according to subscales DC, PD, and P-CDI. The significance level was set at α = 0.05.

## 3. Results

### 3.1. General Characteristics of Participants

Table 2 presents the general characteristics of children with CP and their mothers. The participants were 114 children with CP (mean age = 7.17 years, SD = 2.51, age range = from 2.79 to 11.90). They had been diagnosed by doctors. Male participants constituted 57.0% (n = 65) of the sample, whereas female participants constituted 43.0% (n = 49) of the sample. The age of mothers was 20–39 years old (45.6%), followed by those below 30 years old (14.9%). In terms of educational levels, 94 mothers (82.5%) graduated from university, and 17 mothers (14.9%) graduated from high school.

### 3.2. Unidimensionality

The item fit statistics were shown in Table 3. The statistics were presented based on the order of entry. There were no overfit items (items with an MNSQ value below 0.5) and two misfit items (items with an MNSQ value above 1.7). The first misfit item was number 14, with an infit MNSQ of 1.90 and an outfit MNSQ of 2.02. The second misfit item was number 32, with an infit MNSQ of 1.74 and an outfit MNSQ of 1.96. Across the remaining 34 items, the range of infit MNSQ values was from 0.71 to 1.60, whereas the range of outfit MNSQ values was from 0.69 to 1.56.

### 3.3. Item Difficulty

**Figure 1:**
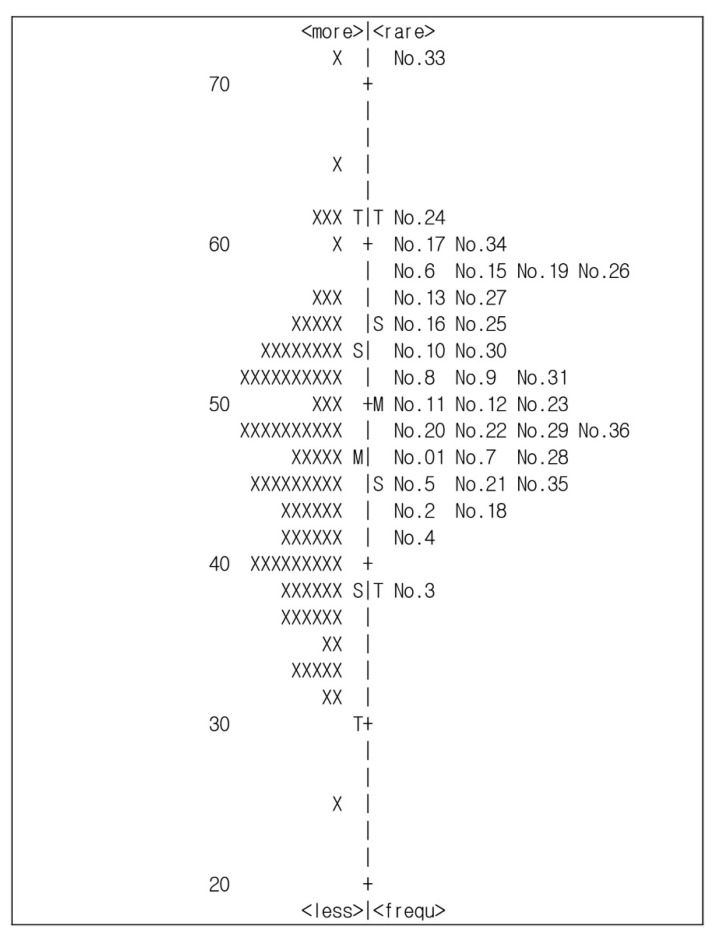
Item difficulty. (Notes: The left map is about person’s ability and the right map is about items’ difficulty. Abler persons and more difficult items are placed on the top of the diagram. # = one persons, M = mean, S = 1 SD from the mean, and T = 2 SD from the mean.).

A map of individual proficiencies and item difficulties for the 34 items of the K-PSI-SF is presented in Figure 1. The most difficult item was Item 33, and the easiest was Item 3. A total of 16 mothers of children with CP exhibited higher proficiency estimates than the ease of Item 3, and a total of two mothers of children with CP exhibited lower proficiency estimates than the difficulty of Item 33.

### 3.4. Separation Index

The person separation reliability value was 0.92, and the separation index was 3.35. The item separation reliability value was 0.95, and the separation index was 4.45 (see Table 4). Thus, both separation indices represented a good level of fit for the K-PSI-SF in mothers of children with CP.

### 3.5. Internal Consistency

The overall stress reliability of the 34-item K-PSI-SF was α = 0.92, the PD reliability was α = 0.90, the P-CDI reliability was α = 0.83, and the DC reliability was α = 0.87(see Table 5). The reliability of the P-CDI and DC subscales, where items with low fit indices were deleted, increased; furthermore, the overall reliability also increased.

### 3.6. Rating Scale Analysis

**Figure 2:**
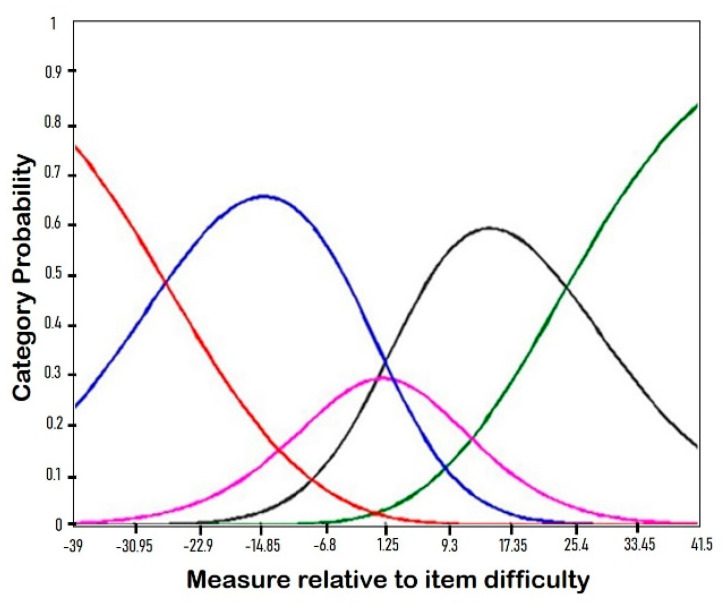
Category probability curve. (Notes: Red line = Category 1; Blue line = Category 2; Pink line = Category 3; Black line = Category 4; Green line = Category 5.).

The results of the 5-point rating scale analysis are presented in Table 6 and Figure 2. This analysis of each aspect of the rating scale indicated that the K-PSI-SF was appropriately adapted for use in mothers of children with CP. The fit statistics were below 1.5 for each response category, and analysis of the scale threshold showed an increase along with increases in the response category.

### 3.7. Parenting Stress Levels across the 36 Items and 34 Items of K-PSI-SF

The mean differences in the K-PSI-SF according to subscales are shown in Table 7. The mean score of the 36 items and 34 items of K-PSI-SF in the PD subscale was same at 2.82 because there was no misfit item in this subscale. The mean score of the P-CDI of 36 items was higher (M = 2.58, SD = 0.54) than that of 34 items (M = 2.48, SD = 0.61), indicating a higher level of parenting stress score when using 36 items of PSI-SF (*t* = 8.635, *p* < 0.001). The mean score of DC of 36 items was higher (M = 2.41, SD = 0.56) than that of 34 items (M = 2.38, SD = 0.57), indicating a higher level of parenting stress when using 36 items of K-PSI-SF (*t* = 3.435, *p* = 0.001).

## 4. Discussion

The purpose of this study was to verity the psychometric properties of K-PSI-SF in mothers of children with CP. The Rasch analysis showed that 34 out of 36 items were an appropriate fit, with an infit MNSQ range from 0.71 to 1.60 and an outfit MNSQ range from 0.69 to 1.56. Two items were not suitable for the mothers of children with CP. One misfit item was number 14. Item 14 belonged to the P-CDI subscale and the question was “Child does not like me or want to be close.” The item fit statistics presented an infit MNSQ of 1.90 and an outfit MNSQ of 2.02. The other item was number 32. Item 32 belonged to the DC subscale and the question was “Getting child to do something is hard.” The item fit statistics presented an infit MNSQ of 1.74 and an outfit MNSQ of 1.96. The infit value is more sensitive to the pattern of responses to items targeted on the person, and the outfit value is more sensitive to responses to items about difficulties far from a person [26]. Those two items were unsuitable based on the characteristics of CP. In a previous study that applied PSI-SF using item response theory (IRT) to parents of children with autism [21], items 14 and 32 were categorized as Category C (poorly functioning items); furthermore, these items were evaluated to be relatively hard to endorse. In Park and Choi’s study [20], the reliability and validity of the K-PSI were not secured for measuring the parenting stress of mothers of children with CP. This was considered to be due to the disability characteristics of children with CP, showing a dysfunction of movement and postures. Because of this, distracted/excessive behavioral questions, such as “active,” “busy,” and “hear and kick,” may be questions that do not reflect the characteristics of children with CP, so it is believed that the reliability was low.

The results of the item difficulty analysis were presented in the form of a figure comparing the locations of parents of children with CP and the locations of items along the latent parental stress dimension. As shown in Figure 1, the range of the respondents’ locations was wider than the range of difficulty levels of the items. Persons at the top of the map indicate the highest level of the measured construct and the items at the top are the most difficult to endorse. When the person mean is higher than the item mean, the items are relatively easy for this sample to endorse. On the other hand, when the person mean is lower than the item mean, the items are considered difficult for this sample to endorse [20]. The range of item difficulty was from 38.30 (Item 3) to 72.31 (Item 33). Additionally, the number of participants with low ability scores (below the easiest item, namely Item 3) was 16 (14.0%), indicating that the items were difficult for mothers of children with CP. Although the results of this study show that the difficulty of the K-PSI-SF is mostly appropriate, consideration must be given to creating easier items for mothers of children with CP. Future research should investigate whether the difficulty of K-PSI-SF is also appropriate for mothers of children with CP.

Rasch analysis provided two kinds of separation index. The person separation reliability value for the K-PSI-SF was 0.92, and the separation index was 3.35; the separation reliability value was 0.95, and the separation index was 4.45. The person separation reliability was considered to be the same as Cronbach’s α. A separation index value above 1.5 implied an acceptable level, and a value above 2.0 showed a good level of the extent of the scale that distinguished each person or item. The separation indices for both person and item separations for the K-PSI-SF were identified as being good. In Lee et al. [23], the total reliability of K-PSI-SF was 0.91 (range from 0.76 to 0.91), which was similar to the value of PSI-SF (range from 0.80 to 0.91).

The rating scale analysis also presented reliable results. Both the infit and outfit of each reply category were below 1.5 [23]. The category level extent for the infit MNSQ was from 0.88 (category level 3) to 1.19 (category level 1) and from 0.85 (category level 3) to 1.11 (category level 1) for the outfit MNSQ. The 5-point scale of the K-PSI-SF revealed the characteristics of the item responses. The probability curve of the K-PSI-SF (Figure 2) showed the appropriateness of the 5-point scale in this regard.

The parenting stress levels across the 36 items and the 34 items of K-PSI-SF were statistically different. After deleting one item each from the P-CDI and DC subscales, each mean of the two subscales became lower. This result means that the two items do not fit into the measurement of the parental stress of mothers of children with CP. Therefore, adjustment of the items may be required. Deater-Deckard and Scarr [29] performed a CFA of the PSI–SF to verify a three-factor model among a sample of parents and reported a poor fit for the PSI-SF. In order to search for a suitable model of the PSI-SF, 17 items were deleted based on the results of EFA, and after confirming the three-factor model through CFA, it was confirmed that the fitness of the PSI-SF was improved.

The K-PSI-SF is valuable as a measure of parenting stress that considers the problems according to the characteristics of infants, as well as the characteristics of infants and young children. Although the development and validation study of K-PSI-SF includes data on parents of children with disabilities, studies on whether it is an appropriate tool for measuring the stress experienced by parents of children with disabilities have been conducted by children’s disability types. However, there were some limitations. The first of these involved sample size. Although we analyzed the data with an adequate sample size, the representativeness of the sample regarding parents of children with CP was limited. Second, degree of disability should have been considered. Parents’ stress level often varies according to the degree of disability [30,31]. Therefore, a psychometric study that considers the degree of disability should be conducted in the future. This is because the parenting stress felt while nurturing infants and toddlers may differ slightly depending on who the subject is. Feasibility studies for general children can also be used to validate PSI-SF for fathers [32] and multi-ethnic parents [33].

## 5. Conclusions

This study confirmed the suitability of the 34 items of K-PSI-SF as a parenting stress measurement tool for children with CP through a Rasch analysis. The 34 questions of K-PSI-SF, which are confirmed to be acceptable indicators of psychometric characteristics, are recommended to be used in future studies to obtain reliable results on parenting stress for mothers of children with CP. This is expected to be used to verify the effectiveness of intervention programs to reduce parenting stress in the mothers of children with CP.

## Figures and Tables

**Table 1:** The contents of the questionnaire (36 items).

Subscale	No. of Items	Cronbach α
Parental Distress (PD)	1~12	0.90
Parent-Child Dysfunctional Interaction (P-CDI)	13~24	0.78
Difficult Child (DC)	25~36	0.83
Total		0.91

**Table 2:** Participants’ Characteristics (children with CP and their mother).

Characteristics	Frequency	%
*Children with CP*		
Gender		
Male	65	57.0
Female	49	43.0
Type of CP		
Spastic	91	79.8
Dystonic	11	9.6
Hypotonic	7	6.1
Ataxic	5	4.4
GMFCS level		
Level 1	35	30.6
Level 2	32	28.1
Level 3	15	13.2
Level 4	13	11.4
Level 5	19	16.7
*Mothers*		
Age		
Below 30	53	14.9
30~39	52	45.6
40~49	7	6.1
Above 50	2	1.8
Education level		
Graduate School	2	1.8
University	94	82.5
High school	17	14.9
Below high school	1	0.9

**Table 3:** Item Fit Statistics.

	Item No.	MEASURE	S.E.	Infit		Outfit	
				MNSQ	Z-Value	MNSQ	Z-Value
Parental Distress	1	45.99	1.11	0.82	−1.6	0.83	−1.4
	2	42.59	1.10	0.82	−1.6	0.82	−1.6
	3	38.09	1.14	0.81	−1.6	0.79	−1.6
	4	41.25	1.11	0.84	−1.4	0.83	−1.4
	5	44.29	1.10	0.71	−2.6	0.72	−2.4
	6	56.13	1.28	0.99	0.0	0.96	−0.2
	7	45.99	1.11	0.75	−2.2	0.73	−2.4
	8	49.90	1.15	0.74	−2.1	0.70	−2.4
	9	50.97	1.16	0.88	−0.9	0.82	−1.3
	10	52.35	1.19	0.81	−1.4	0.80	−1.5
	11	48.36	1.13	0.95	−0.4	0.92	−0.6
	12	49.51	1.14	0.78	−1.8	0.77	−1.8
Parent–Child Dysfunctional Interaction	13	54.40	1.23	1.06	0.5	1.10	0.7
	**14**	**38.74**	**1.13**	**1.90**	**5.7**	**2.02**	**6.1**
	15	56.81	1.31	1.37	2.3	1.34	2.0
	16	52.78	1.20	0.71	−2.3	0.69	−2.4
	17	57.13	1.31	1.40	2.4	1.42	2.5
	18	42.45	1.11	1.33	2.5	1.32	2.4
	19	55.49	1.26	1.59	3.5	1.46	2.7
	20	47.85	1.12	0.82	−1.5	0.79	−1.7
	21	44.05	1.10	0.86	−1.1	0.86	−1.1
	22	47.35	1.12	0.96	−0.3	1.00	0.0
	23	49.23	1.14	0.92	−0.6	0.93	−0.4
	24	58.81	1.36	0.84	−1.0	0.82	−1.1
Difficult Child	25	53.06	1.20	0.88	−0.8	0.84	−1.1
	26	56.79	1.30	1.15	1.0	1.10	0.7
	27	54.34	1.24	0.90	−0.7	0.82	−1.2
	28	46.85	1.11	0.81	−1.6	0.84	−1.2
	29	47.10	1.12	1.02	0.2	1.02	0.2
	30	51.38	1.17	0.86	−1.0	0.86	−1.0
	31	49.90	1.15	1.13	1.0	1.10	0.8
	**32**	**48.11**	**1.13**	**1.74**	**4.9**	**1.96**	**5.8**
	33	72.13	1.79	1.60	3.3	1.56	3.3
	34	57.30	1.31	0.84	−1.0	0.84	−1.0
	35	44.53	1.10	1.05	0.4	1.06	0.5
	36	47.98	1.12	0.75	−2.2	0.73	−2.2

Note: MNSQ = mean square, S.E. = standard error, Misfit values are in bold.

**Table 4:** Person and item separation index of the K-PSI-SF.

Category	Separation Index	Reliability
Person	3.35	0.92
Item	4.45	0.95

**Table 5:** The contents of the questionnaire (34 items).

Subscale	No. of Items	Cronbach α
Parental Distress (PD)	1~12	0.90
Parent-Child Dysfunctional Interaction (P-CDI)	13, 15~24	0.83
Difficult Child (DC)	25~31, 33~36	0.87
Total		0.92

**Table 6:** Rating scale analysis of the K-PSI-SF.

Category level	Observed Count	Average Measure	Infit MNSQ	Outfit MNSQ	Structure Measure
1	273	−12.66	1.19	1.11	NONE
2	1342	−8.59	0.90	0.91	−26.93
3	642	−1.62	0.88	0.85	2.30
4	682	3.82	0.99	1.00	0.19
5	117	10.08	1.02	1.02	24.45

Note: MNSQ = mean square.

**Table 7:** Parenting stress level according to subscale.

Subscales	36 Items		34 Items		*p* Value
	Mean	SD	Mean	SD	
Parent Distress (PD)	2.82	0.72	2.82	0.72	N/A
Parent–Child Dysfunctional Interaction (P-CDI)	2.58	0.54	2.48	0.61	<0.001
Difficult Child (DC)	2.41	0.56	2.38	0.57	0.001

Note: N/A = not applicable.
